# ‘Leading from the front’ implementation increases the success of influenza vaccination drives among healthcare workers: a reanalysis of systematic review evidence using Intervention Component Analysis (ICA) and Qualitative Comparative Analysis (QCA)

**DOI:** 10.1186/s12913-022-08001-6

**Published:** 2022-05-16

**Authors:** Katy Sutcliffe, Dylan Kneale, James Thomas

**Affiliations:** EPPI-Centre, Social Research Institute, UCL Institute of Education, University College London, London, UK

## Abstract

**Background:**

Seasonal influenza vaccination of healthcare workers (HCW) is widely recommended to protect staff and patients. A previous systematic review examined interventions to encourage uptake finding that hard mandates, such as loss of employment for non-vaccination, were more effective than soft mandates, such as signing a declination form, or other interventions such as incentives. Despite these overarching patterns the authors of the review concluded that ‘substantial heterogeneity’ remained requiring further analysis. This paper reanalyses the evidence using Intervention Component Analysis (ICA) and Qualitative Comparative Analysis (QCA) to examine whether the strategies used to implement interventions explain the residual heterogeneity.

**Methods:**

We used ICA to extract implementation features and trialists’ reflections on what underpinned the success of the intervention they evaluated. The ICA findings then informed and structured two QCA analyses to systematically examine associations between implementation features and intervention outcomes. Analysis 1 examined hard mandate studies. Analysis 2 examined soft mandates and other interventions.

**Results:**

In Analysis 1 ICA revealed the significance of ‘leading from the front’ rather than ‘top-down’ implementation of hard mandates. Four key features underpinned this: providing education prior to implementation; two-way engagement so HCW can voice concerns prior to implementation; previous use of other strategies so that institutions ‘don’t-go-in-cold’ with hard-mandates; and support from institutional leadership. QCA revealed that either of two configurations were associated with greater success of hard mandates. The first involves two-way engagement, leadership support and a ‘don’t-go-in-cold’ approach. The second involves leadership support, education and a ‘don’t-go-in-cold’ approach. Reapplying the ‘leading from the front’ theory in Analysis 2 revealed similar patterns.

**Conclusions:**

Regardless of intervention type a ‘leading from the front’ approach to implementation will likely enhance intervention success. While the results pertain to flu vaccination among HCWs, the components identified here may be relevant to public health campaigns regarding COVID-19 vaccination.

**Supplementary information:**

The online version contains supplementary material available at 10.1186/s12913-022-08001-6.

## Introduction

### Seasonal influenza and vaccine hesitancy among healthcare workers

Seasonal influenza can have dire consequences for individuals, particularly for vulnerable groups such as children, older people and those with pre-existing health problems [1]. Outbreaks can also place significant strain on health services [2]. Healthcare Workers (HCWs) involved in direct patient care are encouraged to receive an influenza vaccine [3]. Whilst evidence shows influenza vaccine to be safe, effective, and to decrease mortality in patients [4] a key challenge is poor vaccine uptake. In the 2018–2019 season in England 70% of frontline HCWs were vaccinated, which represents a year-on-year increase, but is short of the national target of 75% [3]. Vaccine hesitancy has been increasing in recent years [5, 6] and the COVID-19 pandemic has highlighted the urgency of understanding how to address it [7], particularly among HCWs to ensure their wellbeing as well as to ensure the delivery of safe, efficient and effective healthcare services [8].

### Current understanding about interventions to increase uptake and gaps in the evidence

A comprehensive systematic review [9], which was recently updated [10], found that various interventions to encourage uptake can increase rates of vaccination among HCW. The review examined both voluntary programmes (such as incentives, media campaigns or education programmes) and policies which make vaccination mandatory for HCWs (of which some were defined as ‘soft’ and others ‘hard’). Meta-analysis was used to quantify the effects of the various approaches in the original review. The findings demonstrated that among the intervention strategies examined, ‘hard’ mandates such as loss of employment for non-vaccination were by far the most effective (RRunvac (risk ratio of being unvaccinated) = 0.18, 95% CI: 0.08–0.45). This was followed by ‘soft’ mandates involving a requirement to be vaccinated accompanied by measures such as requiring staff to sign a declination form, as well as other voluntary interventions such as increasing access (i.e. making it easier for staff to receive the vaccination) (RRunvac = 0.64, 95% CI: 0.45–0.92) and increasing awareness (e.g. through media campaigns) (RRunvac = 0.83, 95% CI: 0.71–0.97). The pooled findings for incentives did not quite reach statistical significance (RRunvac = 0.89, 95% CI: 0.77–1.03) and pooled findings for educational interventions showed no evidence of an effect (RRunvac = 0.96, 95% CI: 0.84–1.10).

Whilst these findings about the pooled effects of interventions within broad categories is a useful step in understanding how best to address the issue of vaccination uptake in HCW, vital knowledge about exactly what to implement and how is lacking. The authors identified ‘substantial heterogeneity’ in the findings ([9] p.66) and acknowledged that this may be due to a number of factors including: the HCW populations studied; the clinical setting; the country; the specific components of each intervention and the way these were implemented in each study. For example, the exact nature of ‘hard-mandates’ varied considerably; some required mask use for unvaccinated HCW whilst others prohibited patient contact and yet others resulted in termination of employment.

Uptake of systematic review findings is often hindered by a lack of information about the specific features and implementation methods of successful strategies [11, 12]. In addition, uptake of this review’s findings may be hindered by ethical concerns about the use of hard mandates suggesting that a more holistic understanding of such strategies is warranted [13].

The high-level findings of the Lytras et al. review about the success of hard-mandates suggest the validity of a ‘sticks are better than carrots’ intervention theory. However, since not all hard-mandate (or soft-mandate) interventions achieved similar rates of success we needed to look beyond the overt intervention theory and to focus on ‘on-the-ground’ implementation and context.

The overarching aim of this research was to support hospitals to implement effective vaccination uptake strategies by identifying the critical features and implementation methods of successful strategies. In addition, by exploring how vaccination uptake strategies work, we hoped to provide some insights that might assist with global drives to vaccinate against COVID-19.

## Materials and methods

The aim of this project was to reanalyse the trials using an alternative analytical technique – qualitative comparative analysis (QCA) [14–16]. QCA – originally developed by Charles C. Ragin in the field of political science [17] – has recently been employed in systematic reviews [18, 19]. When employed in systematic reviews the technique seeks to uncover the key combination of intervention and contextual characteristics associated with a given outcome; underlying causal mechanisms are inferred from the observation of a combination of characteristics and outcome. QCA is a ‘case’ rather than a ‘variable’ oriented approach. A ‘case’ in QCA terms essentially refers to the unit of study. In systematic reviews this is usually an intervention, both its features and the context in which it was implemented. The ‘case’ oriented approach requires a deep and holistic understanding of each case. Another key feature of QCA is that it uses set theory to identify the necessary and sufficient conditions for a particular outcome; for example, this analysis is based on a comparison of the characteristics of a set (i.e. a group) of effective cases as distinguished from sets of ineffective cases. Here, QCA seeks to identify the degree of overlap between these outcome sets and sets of cases with similar intervention or implementation characteristics. This approach enables an analysis that, unlike meta-analysis, can operate with relatively small numbers of studies and a relatively large number of variables (which are referred to as ‘conditions’ in QCA). QCA is also particularly suitable for analysing intervention complexity in systematic reviews because it allows for equifinality; that is, it assumes that different combinations of intervention characteristics may lead to the same outcome. Lastly, whilst some QCA designs employ inductive reasoning or integrate elements of deduction [20], many QCA applications in evidence synthesis adopt an abductive approach which is suited to examining complex causality [21]. Unlike the deductive approach of meta-analysis in which a hypothesis is posed and then tested, the abductive approach involves starting with an observed outcome (in this case rates of vaccination uptake) and working backwards to identify the simplest and most likely explanation for the observed outcome. Because the abductive approach yields a plausible explanation but is not able to conclusively verify it, it is far less secure than a deductive approach. As such a key requirement is that the analysis is underpinned by theory. Implicitly, the application of QCA in systematic reviews differs from that of many other social and political science applications. In the case of systematic reviews, the dataset used as the basis of QCA is one constructed by the reviewers based on the observations and reports of the processes that occurred during the implementation of an intervention. However, given that the adequacy of the reporting of the implementation of interventions is a widespread concern across the literature [12, 22], this can make the treatment of missing data challenging, particularly as triallists almost exclusively report on processes that did occur as opposed to confirming the processes and components that did not occur. Applications of QCA in systematic reviews have assumed that where an intervention process or component is unreported, that it was indeed absent from the intervention [18, 23]. However, to gain a fuller understanding of the processes that did occur within an intervention, we draw on Intervention Component Analysis (ICA).

ICA is a methodological approach which seeks to ‘bridge the gap’ between evidence of intervention effectiveness and practical implementation of interventions [24]. More specifically, ICA seeks to generate an ‘experienced-based’ understanding of intervention mechanisms by tapping into trialists’ informal reflections about how the interventions they evaluated worked ‘on the ground’. ICA uses qualitative data analysis techniques and draws on informal evidence – often reported in the Discussion section of published trial reports – about what trialists’ felt led to the success of an intervention or what inhibited its success. Although there are potential limitations to drawing on informal data of this kind, ICA offers a systematic process through which experience-based theoretical explanations of intervention mechanisms can be developed. These explanations can then be tested using more formal analytical techniques such as QCA. In addition, given that (too) many outcome evaluations fail to be accompanied by a process evaluation, which could provide richer data on intervention mechanisms and fidelity to intervention protocols, ICA provides an alternative framework for incorporating additional data on intervention processes and components. ICA and QCA were paired in a previous project to successfully identify critical intervention mechanisms [25].

The research involved a reanalysis of the trials included in the Lytras et al. 2016 review [9] and from the Lorenc et al. 2018 update [10]. Ethical approval was not obtained since the analysis involved only published data already in the public domain. There are no reporting guidelines for reanalyses of systematic reviews, although guidance for QCA studies is being developed [26] and we have sought to provide a detailed and transparent account of the work such that it could be replicated.

Based on the Lorenc et al. work [27] and an assumption that different interventions would be underpinned by different programme theory, our initial hypothesis was that the mechanisms differentiating the more successful from the less successful hard-mandate interventions would differ from the mechanisms differentiating the more successful of the soft-mandate and other interventions from those that were less successful. Thus, we conducted two separate analyses. Analysis 1 explored which intervention and implementation features were associated with greater effectiveness among the hard-mandate interventions, and Analysis 2 explored which features were associated with greater effectiveness among the soft-mandate and other voluntary interventions. We completed all of the QCA stages for Analysis 1 before repeating the process for Analysis 2.

### QCA stage 0: selection of cases and determining outcome sets

For Analysis 1 we selected all eight of the hard mandate cases [28–34] included in the original review [9] (note: two hard mandate cases were evaluated in the Ksienski 2014 study), and the three additional hard mandate cases [35–37] identified in the update [10]. For Analysis 2 there was a much greater number of non-hard mandate cases (45 cases from the review and 12 from the update) so we were able to purposively select the cases with maximum variation in outcomes, i.e. the 10 most effective non-hard mandate cases [38–45] and the 10 least effective ones [46–52]. (Note: A total of six papers reported the 10 least effective soft mandate / other cases; two cases were reported in each of the following three papers Dey et al. 2001, Doratotaj et al. 2008 and Zimmerman et al. 2009.) By excluding the moderately effective non-hard mandate cases we filtered out ‘noise’ which might obscure differences between the most effective and least effective. Effectiveness was determined as per the original Lytras review in terms of the Relative Risk of remaining unvaccinated after the intervention (RRunvac); values of RRunvac < 1 suggest that the intervention is effective in reducing the number of unvaccinated HCWs. For Analysis 2 we used crisp outcome sets, in which cases are full members of a set of ‘most effective’ cases or full members of a set of ‘least effective cases’. We ranked the cases according to their RRunvac value; the 10 in the most effective set had values ranging from 0.06 to 0.59, the 10 in the least effective set had values ranging from 0.95 to 0.99. Since we included the full range of outcomes for Analysis 1 (i.e. we did not exclude moderately effective cases as we did for Analysis 2) we created fuzzy outcome sets, where studies could be partial members of sets. A highly successful outcome set (coded as 1) comprised of four cases with RRunvac values between 0.01 and 0.14. A moderately successful outcome set (coded as 0.66) comprised of four cases with RRunvac values between 0.15 and 0.29. A least successful outcome set (coded as 0.33) comprised of two cases with RRunvac values between 0.30 and the least effective in the set (0.57). The terminology ‘successful’ for analysis 1 is in relation to other cases included in the model – all studies in Analysis 1 could be considered effective based on their effect sizes and confidence intervals although some were much more impactful than others.

### QCA stage 1: Identification of conditions using ICA and building the data table

Once we had selected our cases and determined our outcome sets we read and re-read the papers reporting the 11 hard-mandate cases to generate a deep knowledge for Analysis 1. After the familiarisation exercise two authors (KS and DK) independently extracted information about the nature of the hard-mandate interventions to create a data table with cases represented in rows and conditions represented in columns (see supporting information – Additional File 1). Initial work focused on the intervention descriptions as provided by the authors – for example we captured data on the nature of hard mandates such as whether it resulted in loss of employment or not, whether there were stigmatising markers of non-identification and whether any ‘declination’ procedures were particularly onerous or not. However, limiting our data collection to the intervention descriptions alone proved unfruitful for identifying features that distinguished between the most and least successful interventions. Thus, we decided to focus on implementation and to employ ICA to extract information from the Discussion section. Specifically, we used inductive qualitative analysis techniques to code authors’ perceptions about the factors that acted as facilitators of or barriers to success. ICA revealed four implementation features that were commonly described by authors as underpinning the success of hard mandate interventions: Education (reported in 5 cases) for example providing information sessions prior to mandate implementation; two-way engagement (reported in 2 cases) i.e. opportunities for HCW to raise concerns; ‘don’t go in cold’ (reported in 5 cases) i.e. efforts in previous years to encourage vaccination uptake; and leadership support (reported in 6 cases) i.e. involvement and endorsement from senior leaders in the institution. Table 1, below provides example statements from authors regarding the importance of these implementation features [53]. We then coded each case to denote the presence and absence of conditions, a condition reported as present was coded as ‘1’ and where a condition was not reported it was assumed to be absent and coded as ‘0’. Before proceeding to the next stages of QCA analysis the quality of the data was evaluated, including checks for ‘collinearity’ of conditions (i.e. when two or more conditions consistently co-occur) and rarity of conditions.

**Table 1:** Example author statements about factors perceived as vital to successful hard-mandate implementation

**Education:** “*Key factors that supported the success of the program included consistent communication emphasizing patient safety and quality of care.*” (Babcock et al. 2010) [29]
**Two-way engagement:*** “Continued stakeholder engagement is required to ensure that the decision-making process is collaborative and the Policy is not viewed as punitive.”* (Ksienski 2014) [30]
**Don’t go in cold:** *“Sequential expansion of the program over several years was a key element to the success.” *(Frenzel et al. 2016) [36]
**Leadership support:** *“Without a strong endorsement from the CEO, president, and governing board, it is unlikely that the program would have been successful.”* (Rakita et al. 2010) [32]

We returned to the theoretical literature to see if existing theories reflecting our emergent findings could help to consolidate our thinking. This process identified the theoretical concept of ‘leading from the front’ as opposed to a ‘top-down’ or ‘authoritarian’ approach to leadership with the key underpinning principle being that organisations should aim to ‘bring people with you’. The concept draws on literature on transformational leadership which emphasises communication, listening, modelling and leadership commitment [54].

The same steps were taken for Analysis 2; however as we had assumed a different mechanism would underpin the non-hard mandate studies we did not initially extract the same conditions as identified in the ICA for Analysis 1. Initial work for Analysis 2 was based on a ‘dark logic’ approach [55]. Since the non-hard mandate interventions were found to be broadly less effective than hard-mandate interventions we considered whether we might identify harmful or ineffective mechanisms that undermined the approach. However, this analytical plan proved unfruitful. So we decided to see if the same conditions and the ‘leading from the front’ theory might also explain the variation in outcomes among the soft-mandate and other interventions.

A concern when conducting QCA is that an imbalance in the ratio of cases to conditions may lead to issues of limited diversity where we do not observe many potential configurations of conditions, and we risk creating an individual description for each case, rather than an explanation based on regularity in patterns. The selection of four conditions for the initial model of hard mandates which contained eleven cases, was based on theoretical assumptions of the key implementation processes as well as guidance of the ideal ratio of cases:conditions. The ratio for the hard mandate model is consistent with common practice and earlier guidance that indicated that between four and seven conditions for an ‘intermediate N’ analysis of between 10 and 40 cases could provide a balance of cases:conditions [53]. Later empirical analyses indicate that our chosen ratio of cases:conditions in the crisp-set hard mandate models is borderline acceptable [56], although we acknowledge that the question on the ideal balance of cases:conditions remains a matter of debate, and is discussed later in the paper.

### QCA stage 2: constructing Truth Tables

In QCA stage 2 a Truth table, the key analytic device of QCA, is created. The Truth Table moves the focus from individual cases to groups of cases sharing the same outcomes ‘outcome sets’ (as described above) and from individual conditions to sets of studies with particular combinations or “configurations” of conditions that lead to a particular outcome. The Truth Tables for analyses 1 (Table 2) and 2 (Table 4) are presented below.

### QCA stage 3: checking the quality of the Truth Tables

The first check of each Truth Table involved assessing the degree to which a consistent pattern of association between the configurations and the outcome sets is evident. For example, if all cases involving all four conditions in the theory (education, two-way engagement, a ‘don’t go in cold’ approach and leadership support) are also all cases that are fully part of the successful outcome set and none are cases in the unsuccessful outcome set, that would show a perfect consistency score, indicated with a ‘1’, for that row of the Truth Table. Conversely, if all cases in which none of the four conditions were present were also all cases in the unsuccessful outcome set, this would also show perfect consistency and be indicated by a ‘0’. Some level of inconsistency is permitted and even expected with fuzzy-set QCA (our consistency score was set at 0.85) – but patterns of association should be evident, and inconsistency explored for potential deviant cases; for crisp-set QCA, consistency scores are expected to be as close to 1 as possible (perfect consistency) [56] and here, given the relatively small size of the dataset, we set our consistency level at one, so that any deviation or inconsistency needs to be resolved or explained. The second check we performed was to assess coverage, i.e. whether configurations are supported by multiple cases. It is expected that there will be several paths to a given outcome, and so the coverage offered by any given configuration may only be one or a small number of cases. However, where multiple cases support a configuration - it helps us to understand the relevance or importance of different configurations, and reduces the possibility that the resulting QCA solution becomes an explanation of individual cases. A third check examined whether there was a reasonable spread of cases across the possible configurations in each of our truth tables. Having evidence for a range of possible configurations helps us to interpret and refine our causal theory. Final checks included (i) examining for deviant cases consistency [57] - those cases with values above 0.5 for the condition configuration and below 0.5 for the outcome (inconsistencies); and (ii) examination of counterintuitive findings – e.g. if cases with all conditions specified in our underlying theory were associated with unsuccessful outcomes – indicating that our theory does not play out in practice. As the Truth Tables below illustrate, we found satisfactory results for each of the above checks.

### QCA stage 4: Boolean minimization to identify simplified expressions of configurations

We used Boolean minimisation to identify simplified expressions of configurations, with variants in how Boolean minimisation was conducted based on the treatment of logical remainders (see Stage 5). Configurations were simplified with view of maximising coverage of as many of the cases in the successful outcome set as possible and with high consistency. This initially generated what is known in QCA parlance as a ‘conservative solution’ (also known as the complex solution i.e. the longest, least parsimonious solution), where no logical remainders were used in the Boolean minimisation process to simplify the expressions.

### QCA stage 5: consideration of “logical remainders”

In this stage possible configurations for which no cases are available (known as logical remainders) are used to assist with producing a simplified QCA solution (known as the ‘parsimonious solution’). Software (an R package developed by Duşa (2019)) [57] was used to impute outcomes for logical remainders, and this information was accounted for in the QCA solutions, initially generating what was known as a parsimonious solution. The ‘parsimonious solution’ involves the use of an algorithm to assign outcome OR non-outcome to the remainder configurations in such a way as to maximise parsimony. However, in obtaining this solution, some untenable assumptions may have been made in the interest of parsimony, and we generated a further ‘intermediate solution’ that incorporated our own assumptions about the impact of different components (all assumed to be positive in generating a successful outcome). Furthermore, we implemented an algorithm developed by Duşa (2019) to remove untenable and contradictory logical remainders that could be otherwise be used to generate the solution, generating an ‘enhanced intermediate solution’ [58]. This solution represented our preferred solution, and is the basis of our interpretation in the results.

### QCA stage 6: interpreting the solutions

Once we had our QCA solutions we returned to our cases and theory to check that the solutions made sense in the context of individual cases and across cases as a general explanation.

## Results

### Hard mandate studies

QCA revealed that the ‘leading from the front’ theory appeared to explain why some hard-mandate interventions were more successful than others. As the Truth Table (Table 2) below, based on fuzzy-set data, illustrates we had cases for five of the 16 possible configurations. The table illustrates that there is perfect consistency in the relationship between the configuration with all four conditions and cases with the highest levels of vaccine uptake (top row). There is also perfect consistency between higher rates of vaccine uptake and the configuration in which education was absent from the intervention, but the other three conditions were present – although there was only one case with this configuration (second row). The table shows high consistency (0.855) with successful outcomes for the configuration with no two-way engagement but the other three conditions present (row 3, 2 cases). The final two rows illustrate the relationship between configurations associated with unsuccessful outcomes. A configuration in which no intervention components of interest were present, was found in three cases deemed to be mainly unsuccessful and one partially successful case, while a configuration with two components was found in one mainly unsuccessful case. We also emphasise that all the studies achieved statistically significant reductions in the risk of HCWs remaining unvaccinated, and the language of ‘successful’ and ‘unsuccessful’ is relative rather than absolute in this set of results.

**Table 2:** Truth Table for hard mandate QCA (*n* = 11 cases)

Conditions				Outcome	Number of Studies	Consist-ency	PRI	cases
Two-way engage-ment	**Strong Leader-ship Support**	**Edu-cation compo-nent**	**Don’t go in cold**					
1	1	1	1	1	3	1	1	Babcock, Rakita, smith
1	1	0	1	1	1	1	1	Stuart
0	1	1	1	1	2	0.855	0.795	Drees, Frenzel
0	0	0	0	0	4	0.45	0.137	Awali, Ksienski A (Hospital), Ksienski B (ResiCare), Leibu
0	1	0	1	0	1	0.33	0.00	Podscervinsci

*PRI* Proportional Reduction in Inconsistency – a measure of how well a configuration distinguishes between the outcome and its negation

Boolean minimisation, and the generation of an enhanced intermediate solution identified one model containing two simplified pathways of hard mandate implementation that lead to greater vaccination uptake as illustrated in Table 3 below. The first involves two-way engagement, leadership support and a ‘don’t-go-in-cold’ approach. The second involves leadership support, education and a ‘don’t-go-in-cold’ approach. Therefore, an intervention containing either configuration of components and processes is sufficient to result in a successful outcome. Since both pathways contain leadership support and a ‘don’t go in cold approach’ we can infer that these two conditions are necessary but not sufficient to trigger a positive outcome, a third condition is also required. Both configurations cover the majority of instances of the outcome, and crucially they contain all the studies identified as full members of the ‘successful’ outcome set.

**Table 3:** Minimised intermediate solution for hard mandate QCA

Pathway/ Model	Minimised pathway descriptions (see notes)	Consistency	PRI	Raw Coverage	Unique Coverage	cases
1	TWOWAYENG*LEADSUP *DONTGOCOLD	1	1	0.408	0.137	Stuart; Babcock, Rakita, Smith
2	LEADSUP *EDUC *DONTGOCOLD	0.915	0.897	0.499	0.227	Drees, Frenzel; Babcock, Rakita, Smith
M1		0.932	0.921	0.636		
TWOWAYENGAGE*LEADSUP *DONTGOCOLD + LEADSUP*EDUC*DONTGOCOLD = > SUCCESS						

Condition labels: TWOWAYENG = Two-way engagement; LEADSUP = Leadership support; EDUC = Educational component; DONTGOCOLD = Don’t go in cold. Upper case conditions indicate the condition is present and lower case indicate a condition is absent; * = ‘AND’ relationship; + = ‘OR’ relationship; Raw coverage: share of outcome covered by a configuration; Unique coverage: share of outcome uniquely coverage by a configuration. Only one model was generated as a solution (M1) with two pathways, suggesting no model ambiguity

### Soft mandate / other studies (*n* = 20 cases)

The Truth Table below (Table 4) presents configurations using the same four conditions as specified in the ‘leading from the front’ theory, plus an additional condition ‘letter only’. When we first assessed the 20 soft mandate / other cases we had trouble understanding why some highly effective studies did not fit with the theory. It is possible that there are other conditions or contextual factors that explain their success. However, we noticed that these particular studies contained scant information as they were not full research papers but letters only (i.e. much shorter in length than a standard article), albeit containing empirical information; in particular, they had limited Discussion sections which is where critical information, for example about the influence of strong leadership support, was generally reported. Thus, we made the assumption that some of the critical features in the theory could be present in these cases but just not described due to the shorter length of this type of article. Once we coded cases as ‘letter only’ (or research articles) and included this in the model, the same patterns began to emerge.

For example, the Truth Table makes clear that all but one of the configurations associated with least effectiveness – in the six bottom rows - did not involve strong leadership support. In contrast, all cases associated with greater effectiveness (aside from two which were letters only) did involve leadership support. Similarly, all cases bar one identified as having a successful outcome had evidence of activities being implemented before the intervention; the one case that did not was a letter.

**Table 4:** Truth Table for Soft Mandate / Other QCA (*n* = 20 cases)

Strong Leader-ship Support	Don’t go in cold	Two-way engage-ment	Edu-cation Com-ponent	Letter	Outcome	Number of Studies	Consistency	PRI	Cases
1	1	1	1	0	1	4	1	1	Thomas, Lavela, Heinrich, LeMaitre
0	0	0	1	1	1	1	1	1	Shannon
0	1	1	1	1	1	1	1	1	Sadlier
1	1	0	1	0	1	1	1	1	Ribner
1	1	0	1	1	1	1	1	1	Lopes
1	1	1	0	0	1	1	1	1	Honda
1	1	1	1	1	1	1	1	1	Guanche Garcel
0	1	0	1	0	0	4	0	0	Doratoraj a (letter), Camarago, Zimmerman a (incentives), Zimmerman b (increased access)
0	0	1	1	0	0	2	0	0	Dey a, Dey b
0	0	0	1	0	0	1	0	0	Leitmeyer
0	1	0	0	0	0	1	0	0	Doratoraj b (raffle)
0	1	1	1	0	0	1	0	0	Smedley
1	0	0	1	0	0	1	0	0	Rothan-Tondeur (educ only)

*PRI *Proportional Reduction in Inconsistency – a measure of how well a configuration distinguishes between the outcome and its negation

**Table 5:** Minimised intermediate solution for soft mandate / other QCA

Pathway/ Model	Minimised pathway descriptions (see notes)	Consist-ency	PRI	Raw Coverage	Unique Coverage	cases
1	LEADSUP*DONTGOCOLD*EDUCATION	1	1	0.7	0.3	Ribner; Lopes; Thomas, Lavela, Heinrich, LeMaitre; Guanche Garcel
2	LEADSUP*DONTGOCOLD* TWOWAYENG*letter	1	1	0.5	0.1	Honda; Thomas, Lavela, Heinrich, LeMaitre
3	leadsup*EDUC*LETTER	1	1	0.2	0.2	Shannon; Sadlier
M1		1	1	1		
leadsup*EDUC*LETTER + LEADSUP*DONTGOCOLD*EDUCATION + LEADSUP*DONTGOCOLD*TWOWAYENG*letter = > SUCCESS						

Condition labels: LETTER = Letter publication; see Table 3 for other condition names; Upper case conditions indicate the condition is present and lower case indicate a condition is absent; * = ‘AND’ relationship; + = ‘OR’ relationship; Raw coverage: share of outcome covered by a configuration; Unique coverage: share of outcome uniquely coverage by a configuration. Only one model was generated as a solution (M1) with two pathways, suggesting no model ambiguity

Boolean minimisation, and the generation of an enhanced intermediate solution identified one model containing three simplified pathways of soft mandate and other intervention implementation that led to greater vaccination uptake as illustrated in Table 5 above. These mirror the elements in the solution for hard mandates, with the first two pathways indicating that a combination of ‘leadership support’ and ‘don’t-go-in-cold’ is necessary but not sufficient for triggering positive outcomes. In the first pathway, an additional condition for education was part of the configurations, with the seven studies featuring in this pathway representing a mixture of letters and research articles. In addition to ‘leading from the font’ and ‘don’t-go-in-cold’, the second pathway also includes a condition that is complex to capture within a letter – two way engagement – and unsurprisingly all five cases supporting this pathway were reported in full research articles. The third configuration involved two studies, reported as letters only, with additional conditions representing the absence of reported leadership support and the presence of education. This third pathway consists of two studies where the narrow confines of a letter are unlikely to have allowed for more complex mechanisms and processes such as ‘leaderships support’, two-way engagement, and ‘don’t go in cold’. The data in this QCA model were crisp-set, which facilitated the identification of all instances of the outcome (coverage value of 1) with a coverage score of 1.

Having developed familiarity with the framework and the conditions, we then examined the hard mandates using the crisp-set coding framework developed for the soft mandate/other intervention analysis, and distinguishing those four studies with a RR (< 0.2) as (most) successful. Working through the same procedures as the earlier analyses, an enhanced intermediate solution was generated that once again emphasised the importance of the combination of three conditions ‘leading from the front’, ‘don’t go in cold’ and ‘two-way engagement’ as sufficient for generating a successful intervention (Table 6).

Further checks on the solutions represented in Tables 3, 5 and 6 were undertaken. These showed that the solutions did not also trigger the negation of the outcome (a possibility in QCA due to causal asymmetry), and the enhanced intermediate solution generated, using the algorithm developed by Dusa (2019), ensured that untenable simplifying assumptions were not included in deriving our preferred solution.

**Table 6:** Minimised intermediate solution for crisp-set hard mandate QCA

Pathway/ Model	Minimised pathway descriptions (see notes)	Consistency	PRI	Raw Coverage	Unique Coverage	cases
1	TWOWAYENG*LEADSUP*DONTGOCOLD	1	1	1	-	Stuart; Babcock, Rakita, Smith
M1		1	1	1		
TWOWAYENG*LEADSUP*DONTGOCOLD = > SUCCESS						

See Table 3 for condition names; Upper case conditions indicate the condition is present and lower case indicate a condition is absent; * = ‘AND’ relationship; + = ‘OR’ relationship; Raw coverage: share of outcome covered by a configuration; Unique coverage: share of outcome uniquely coverage by a configuration

## Discussion

The above findings reveal that a ‘leading from the front’ rather than a ‘top-down’ approach enhances the effectiveness of flu vaccination drives to increase uptake among HCW. Interestingly, this approach seems to enhance the effectiveness of both hard-mandate approaches and soft-mandates or other approaches. These findings are particularly striking given that the ‘leading from the front logic’ appears to be somewhat in contrast with the overt intervention logic of hard mandates being ‘sticks’ or sanctions to enforce compliance with vaccination drives. By revealing this more nuanced take on hard mandate approaches, our analyses provide additional support for organisations seeking to implement compulsory vaccination drives. Moreover, without this nuanced understanding of key implementation and contextual factors hard mandate approaches may become ineffective in the longer term. We may also expect the lessons learnt from these analyses on flu vaccination uptake to have broader current relevance given the twin global concerns of vaccine hesitancy and COVID-19.

Strategies to vaccinate HCWs against infectious diseases have been thrown into sharp relief by the COVID-19 pandemic and the large-scale efforts to vaccinate HCWs against the SARS-CoV-2 virus taking place across countries. Achieving success in campaigns to vaccinate HCWs is of paramount importance as a means of reducing transmission of the virus to vulnerable patients and in order to protect HCWs due to their increased exposure. However, success in vaccinating HCWs is also likely to have broader implications in terms of vaccination uptake, due the influence of HCWs in decisions about vaccination uptake among the general population [59]. The components highlighted here suggest that successful vaccination campaigns among HCWs are dependent on complex conditions, including ‘don’t-go-in-cold’, ‘two-way engagement’ and ‘leading-from-the-front’. Rather than being aligned with any particular model or specific components or activities, these conditions could be considered design principles to be incorporated into future vaccination campaigns. These conditions may also have some salience in considering wider pandemic control measures. In the UK context for example, which at the time of writing (October 2021) has one of the highest death rates of any large country [60], explanations put forward for non-adherence to pandemic control measures among the general population have parallels with the conditions identified here. For example, the high-profile breach of stay-at-home and social distancing requirements by Dominic Cummings, the Prime Minister’s special advisor, and the subsequent defence of his actions by members of the UK cabinet, has been attributed to weakening adherence to the rules among the population [61] and there are similar concerns about the impact of parties held at the Prime Minister’s residence in contravention of public health regulations [62]; as well as the efforts of UK Imams to counter vaccine hesitancy among the UK’s Muslim population [63] can all be viewed as emblematic of ‘leading-from-the-front’.

### Strengths and limitations

This study presents several innovations that help to advance the use of QCA as an evidence synthesis method. First, the QCA drew on a theory developed from the observations of trialists themselves, from the ‘ground up’ and akin to a grounded theory approach. Previous QCA syntheses of systematic review findings have either necessitated drawing on intervention theories derived from logic models with syntheses of process evaluation studies [64], or other separate in-depth qualitative evidence syntheses [19]. The findings here suggest that, in the absence of extant intervention theory or pre-existing synthesis, that working/pragmatic theories can be developed to support QCA synthesis from experiential evidence that is usually overlooked in other synthesis methods, using an ICA framework. Second, this study showed that a theory of how interventions ‘work’, developed through the synthesis of one set of studies using QCA (i.e. the hard mandate studies), can be applied to a conceptually congruent set of separate studies (i.e. the soft mandate and other intervention studies). This form of triangulation can represent a useful adjunct to QCA analyses in systematic reviews that could help to create more robust syntheses in the future. Third, the study also provided a comparison between using fuzzy-set and crisp-set coding schema on the same dataset (hard mandate studies). While similar results were obtained, again providing a further degree of triangulation, the fuzzy-set coding for the hard mandate studies was a more appropriate choice conceptually. This was with respect to both the coding for the outcome, where all the studies had obtained significant reductions in unvaccinated (despite heterogeneity in the original meta-analysis [9]), as well as the conditions, where in the case of ‘don’t go in cold’ in particular, different levels of previous engagement were apparent among some hard mandate studies in a way which wasn’t as apparent for studies on soft mandates and other intervention modes. Fourth, this is the first example that we are aware of where ‘publication type’ was included in the analysis and was predictive of outcomes. This work thus provides some evidence in support of one issue that’s been long suspected in systematic reviews: that the lack of information in some papers / publications can lead to unreliable review results – and possibly undermine other subgroup analyses [22]. Finally, this study once again is further demonstration of the potential for further adjunct analysis of evidence that has already been assembled and synthesised in some form, to address new questions and generate new understandings. This study drew on ICA/QCA; other techniques for the reanalysis of existing review evidence have also been suggested elsewhere [65]. Given the large volume of systematic reviews being published annually, each requiring substantial investment and sometimes generating conflicting results or interpretations, techniques for further probing of the included studies to provide additional nuance or address questions not considered by the original reviewers, may continue to develop as a promising adjunct stream of evidence synthesis.

While the analyses presented here are of importance, both in (i) revealing some of the conditions sufficient to result in successful influenza vaccination campaigns: as well as (ii) emphasising the potential of ICA/QCA in enhancing our understanding of existing review evidence, some limitations should be noted. An important limitation is around the approach itself and its capacity to consistently and correctly reveal complex causal relationships. There exist some critiques around the potential of QCA to produce correct solutions in simulated data sets for which true causal processes are known [66], although responses provided by others not only highlight flaws in these critiques, but also emphasise that a QCA solution cannot be generated and articulated in the absence of case and substantive knowledge [67]. While we regard the use of ICA to generate theory to underpin QCA as a useful innovation in the field; we nevertheless recognise that trial reports remain sparse in terms of reporting intervention details [12], and despite the allowances we made for sparse reporting in letters, ‘missing data’ may be a further caveat on the results. However, a strategy analogous to a ‘‘complete case” approach sometimes used in statistical analysis, where only cases reporting either the presence or absence of a condition are included in the analysis, would not have been appropriate here and would have led to no viable cases being identified. Instead, we have assumed that where a process or intervention component was not reported, that it did not take place; this is a strategy mirrored across the whole of the systematic reviewing literature. The inclusion of a condition for article type (with a letter indicating a short empirical report) reflects that some article types are constrained in the detail that can be published such that some processes that did take place are at risk of going unreported. This is not a marker of study quality per se but of reporting quality and was used in this case to understand and explain why some cases with a low number of active components and processes were successful (contrary to expected theory). The inclusion of the reporting style as a condition does represent a potential limitation as it requires a different interpretation to the other conditions (explanatory not causal). Another potential limitation is the relatively low ratio of cases per conditions, particularly for the hard mandates model, which generated a number of logical remainders which could undermine the soundness of our conclusions [56]. We based our chosen conditions on our working theory of how the intervention was expected to work, and selected the number of conditions based on guidance proposed on the ideal balance of cases:conditions [53], which was later investigated more thoroughly [56]. The results of investigations conducted by Marx and Dusa [56] suggest that while the number of conditions (4) is relatively high for an intermediate dataset of 11 studies, it remains on the borderline of acceptability. Nevertheless, limited diversity and the relatively high number of unobserved potential configurations is a limitation of this model in particular. [53]Finally, while we generated an enhanced intermediate solution as proposed by Schneider and Wagemann [58], and following procedures developed by Duşa [57], the treatment of logical remainders somewhat contested and unresolved in the literature [68, 69]. Thus, even though some have suggested a growing consensus in support of the prioritization of intermediate solutions [70, 71], this could represent a final caveat to these results. However, since QCA requires that the solution is consistent with a programme theory that is identifiable in all relevant cases, it can be seen, in some ways, as having a higher bar for achieving a credible explanation than statistical analysis. In a statistical analysis, deviant cases might increase variance / widen confidence intervals, but are considered ‘explained’ when this happens. In a QCA, a deviant case indicates that a credible solution that properly explains what is going on has not be found, so further analysis is required. As such, given that we identified consistent patterns of association across several independent research studies and that the detail of each case was consistent with our ‘leading from the front’ theory, the credibility of these findings is strengthened.

## Conclusions

Regardless of intervention type a ‘leading from the front’ approach to implementation, which incorporates building on institutional knowledge, education, opportunities for two-way engagement and strong leadership support, will likely enhance the success of HCW flu vaccination drives. While the results pertain to flu vaccination and HCW populations, the nuanced understanding of effective intervention strategies identified here may be useful in the urgent efforts to vaccinate HCW and the general public against COVID-19.

## Supplementary information

**Additional file 1.**

## Data Availability

All data analysed for this study are available in the published studies included in the analysis. Additional File 1 contains the information extracted into the data tables that were used to perform the analyses.

## Abbreviations

HCW: Healthcare worker
ICA: Intervention Component Analysis
QCA: Qualitative Comparative Analysis
RRunvac: Risk ratio of being unvaccinated
