# Supplementary material for: ‘Leading from the front’ implementation increases the success of influenza vaccination drives among healthcare workers: a reanalysis of systematic review evidence using Intervention Component Analysis (ICA) and Qualitative Comparative Analysis (QCA)
Source: BMC Health Serv Res. 2022 May 16;22:653. doi: 10.1186/s12913-022-08001-6 (PMC9108687; doi:10.1186/s12913-022-08001-6)
Supplement: Supplementary file 1 — Additional file 1. [file 12913_2022_8001_MOESM1_ESM.docx]

Additional File 1

Supplementary Table 1: Hard mandate (Analysis 1) Fuzzy set Data table

| Studies | Outcome set | Don’t go in cold (DONTGOCOLD) | Educa-tion Comp-onent (EDUC) | Inform on other (INFORM)* | Two-way engage-ment  (TWOWAYENG) | Stigma (STIGMA)* | Strong Leader-ship Support  (LEADSUP) | Multi-setting (MULTI)* |
| --- | --- | --- | --- | --- | --- | --- | --- | --- |
| Awali | 0.33 | 0.33 | 0 | 0.66 | 0 | 0 | 0 | 0 |
| Babcock | 1 | 1 | 1 | 0 | 0.66 | 0 | 1 | 0.66 |
| Drees | 0.66 | 1 | 1 | 0.66 | 0 | 1 | 0.66 | 0.33 |
| Frenzel | 0.66 | 1 | 1 | 0.66 | 0 | 0.66 | 1 | 0.33 |
| Hospital-Ksienski | 0.33 | 0 | 0 | 1 | 0 | 0.66 | 0 | 1 |
| ResiCare-Ksienski | 0.33 | 0 | 0 | 1 | 0 | 0.66 | 0 | 1 |
| Leibu | 0.66 | 0 | 0 | 0.33 | 0 | 0 | 0 | 0.66 |
| Podscervinsci | 0.33 | 1 | 0 | 0.66 | 0 | 0.33 | 1 | 0 |
| Rakita | 1 | 0.66 | 1 | 0.66 | 1 | 0 | 1 | 0.66 |
| smith | 1 | 0.66 | 1 | 0.66 | 0.66 | 0 | 1 | 0.66 |
| stuart | 1 | 1 | 0 | 0.33 | 1 | 0 | 1 | 0.33 |

*Not included in final models

Supplementary Table 2: Soft mandate/other (Analysis 2) Data table

| Study | Successful | Stigma (STIGMA)* | Strong Leadership Support  (LEADSUP) | Don’t go in cold (DONTGOCOLD) | Two-way engagement  (TWOWAYENG) | Inform on other (INFORM)* | Education Component (EDUC) | Multi-setting (MULTI)* | letter only |
| --- | --- | --- | --- | --- | --- | --- | --- | --- | --- |
| Dey | 0 | 0 | 0 | 0 | 1 | 0 | 1 | 1 | 0 |
| Doratoraj letter | 0 | 0 | 0 | 1 | 0 | 0 | 1 | 0 | 0 |
| Smedley | 0 | 0 | 0 | 1 | 1 | 0 | 1 | 0 | 0 |
| Camarago | 0 | 0 | 0 | 1 | 0 | 0 | 1 | 0 | 0 |
| Rothan-Tondeur (educ only) | 0 | 0 | 1 | 0 | 0 | 0 | 1 | 1 | 0 |
| Zimmerman incentives | 0 | 0 | 0 | 1 | 0 | 0 | 1 | 0 | 0 |
| Zimmerman increased access | 0 | 0 | 0 | 1 | 0 | 0 | 1 | 0 | 0 |
| Leitmeyer | 0 | 0 | 0 | 0 | 0 | 0 | 1 | 1 | 0 |
| Dey b | 0 | 0 | 0 | 0 | 1 | 0 | 1 | 1 | 0 |
| Doratorajb - raffle | 0 | 0 | 0 | 1 | 0 | 0 | 0 | 0 | 0 |
| Lopes | 1 | 0 | 1 | 1 | 0 | 0 | 1 | 0 | 1 |
| Ribner | 1 | 0 | 1 | 1 | 0 | 0 | 1 | 0 | 0 |
| Shannon | 1 | 0 | 0 | 0 | 0 | 0 | 1 | 0 | 1 |
| Thomas | 1 | 0 | 1 | 1 | 1 | 0 | 1 | 0 | 0 |
| Lavela | 1 | 0 | 1 | 1 | 1 | 0 | 1 | 0 | 0 |
| Heinrich | 1 | 1 | 1 | 1 | 1 | 1 | 1 | 0 | 0 |
| LeMaitre | 1 | 0 | 1 | 1 | 1 | 0 | 1 | 1 | 0 |
| Honda | 1 | 0 | 1 | 1 | 1 | 0 | 0 | 0 | 0 |
| Guanche Garcel | 1 | 0 | 1 | 1 | 1 | 0 | 1 | 0 | 1 |
| Sadlier | 1 | 0 | 0 | 1 | 1 | 0 | 1 | 0 | 1 |

*Not included in final models

Supplementary Table 3: Hard mandate (Analysis 1) Crisp set Data table

| Studies | RR | Out-come set | Don’t go in cold (DONTGOCOLD) | Education Com-ponent (EDUC) | Two-way engagement  (TWOWAYENG) | Strong Leader-ship Support  (LEADSUP) |
| --- | --- | --- | --- | --- | --- | --- |
| Awali | 0.35 | 0 | 1 | 0 | 0 | 0 |
| Babcock | 0.06 | 1 | 1 | 1 | 1 | 1 |
| Drees | 0.24 | 0 | 1 | 1 | 0 | 1 |
| Frenzel | 0.21 | 0 | 1 | 1 | 0 | 1 |
| Hospital-Ksienski | 0.44 | 0 | 0 | 0 | 0 | 0 |
| ResiCare-Ksienski | 0.57 | 0 | 0 | 0 | 0 | 0 |
| Leibu | 0.22 | 0 | 0 | 0 | 0 | 0 |
| podscervinsci | 0.42 | 0 | 1 | 0 | 0 | 1 |
| Rakita | 0.05 | 1 | 1 | 1 | 1 | 1 |
| smith | 0.08 | 1 | 1 | 1 | 1 | 1 |
| stuart | 0.14 | 1 | 1 | 0 | 1 | 1 |

## Supplementary Table 4: Hard Mandate (Analysis 1) Crisp Set Truth table

| Two-way engage-ment  (TWOWAYENG) | Strong Leader-ship Support  (LEADSUP) | Educat-ion Component (EDUC) | Don’t go in cold (DONTGOCOLD) | Out-come | Number of Studies | Consistency | PRI | cases |
| --- | --- | --- | --- | --- | --- | --- | --- | --- |
| 1 | 1 | 1 | 1 | 1 | 3 | 1 | 1 | Babcock, Rakita, Smith |
| 1 | 1 | 0 | 1 | 1 | 1 | 1 | 1 | Stuart |
| 0 | 0 | 0 | 0 | 0 | 3 | 0 | 0 | Ksienski A (Hospital), Ksienski B (ResiCare), Leibu |
| 0 | 1 | 1 | 1 | 0 | 2 | 0 | 0 | Drees, Frenzel |
| 0 | 0 | 0 | 1 | 0 | 1 | 0 | 0 | Awali |
| 0 | 1 | 0 | 1 | 0 | 1 | 0 | 0 | Podscervinsci |

Supplementary Table 5: Hard mandate data table with evidence

|  |  |  |  | **‘Don’t go in cold’ (Have there been other previous vaccination campaigns prior to current hard mandate intervention to change behaviours)** | **Was education provided to improve knowledge among HCW of vaccination benefits and risks?** | **Were Healthcare Workers encouraged to inform on others?** | **Two-way engagement/bidirectional communication** | **Stigma** | **Area policies implemented across institutions** | **Leading from the front – did senior staff engage in the campaign** |
| --- | --- | --- | --- | --- | --- | --- | --- | --- | --- | --- |
| **Studies** | **Risk Ratio** | **Percentage of HCWs vaccinated at the end of intervention** | **Outcome set value** | **0 – No effort mentioned**  **0.33 – Sanction-based or accountability-based efforts only previously**  **0.66 – Incentivisation and health promotion based efforts previously**  **1 – Combination of sanction and incentive-based measures used prior to current intervention** | **0 – No education provided or education or education did not improve levels of knowledge**  **1 – Education provided that improved knowledge of the vaccine** | **1 – Employees encouraged to inform on colleagues**  **0.66 – Managers observed compliance**  **0 – No evidence of policy implemented across all employees** | **0 - not stated**  **0.66 - engagement not continuously sought and no formal system - ad hoc**  **1 - formal processes for two way engagement established** | **0 – not stated**  **0.33 – stigmatising process of declination**  **0.66 – public display of vaccination status enforced only**  **1 – stigmatising language with public display of vaccination status** | **0 – single institution**  **0.33 – multiple institutions and/or number of employees >1,500**  **0.66 – multicentre institutions**  **1 – Area based interventions (e.g. states or counties)** | **0 – not stated**  **0.66 – leadership approved and facilitated campaign**  **1 –leadership engaged in and publically voice support for vaccination campaign OR described as being instrumental for success** |
| **Awali** | **0.35** | **93** | **0.33** | **Value: 0.33;** Previous efforts were sanction based only “‘During the next season (2010-2011), the institutional vaccination policy was not strictly mandatory; however, all unvaccinated employees were required to wear masks when within 6 feet of all patient contacts.” | **Value: 0**; “The reluctance of some HCP at our hospital to receive the influenza vaccine despite the mandatory vaccination policy most likely reflects misperceptions and poor knowledge of the benefits and risks of the vaccine. “ | **Value: 0.66**; Managers monitored compliance “The OHS and HCP’s direct supervisors or managers are responsible for ensuring compliance with this policy.” | **Value 0**; No evidence | **Value 0**; No evidence of stigmatising processes | **Value 0**; “…a cross-sectional survey research study was conducted at an urban tertiary care hospital in the metropolitan Detroit area” | **Value 0;** not stated |
| **Babcock** | **0.06** | **98.4** | **1** | **Value: 1;** Non-sanction based campaigns initially before accountability-based measures imposed: “free vaccine available at multiple sites and times, extensive publicity, incentives and educational programs, and more recently, declination statements….. In 2007, influenza vaccination rates were added to the BJC patient safety and quality scorecard used at all hospitals in the organization….. In 2008, BJC HealthCare implemented a mandatory influenza vaccination policy for all employees” | **Value: 1**; “Key factors that supported the success of the program included consistent communication emphasizing patient safety and quality of care, coordinated campaigns, leadership support, and medical director support to talk with any employee with concerns about the vaccine, on request.” | **Value: 0**; No specific enforcement or data on compliance collected | **Value 0.66**; Ad hoc interactions: "Managers interacted with their staff to ascertain reasons for noncompliance and to provide coaching about influenza” | **Value 0**; No evidence of stigmatising processes; encouraged to wear mask | **Value 0.66**; “Facilities include 11 acute care hospitals and 3 extended care facilities, as well as day care centers, employed physician groups, occupational medicine, home care, and behavioral health services.” | **Value 1; “**The CEO of BJC published a letter in the BJC newspaper explaining the rationale for the policy. The multidisciplinary implementation team met regularly before and during the vaccination campaign to ensure timely, consistent, and coordinated communication and responses to any issues that arose.” |
| **Drees** | **0.24** | **92** | **0.66** | **Value: 1;** Non-sanction based campaigns initially escalating to other forms of mandates/sanctions: “[the] vaccination campaign included promotional materials, web-based and in-person education, free vaccination for employees and medical-dental staff, roving vaccinators…During the 2009 H1N1 pandemic, the health system created a policy that required explicit declination by all employees as well as the wearing of surgical masks….However, the policy did not include provisions to enforce either of these measures.” | **Value: 1**; from supplementary materials, the communication campaign included: “Launched internal flu website with explanation of new program, frequently asked questions, multiple resources and links to external sources; Web-based education (non-mandatory) to all employees” | **Value: 0.66**; Managers monitored compliance “Beginning 2 weeks after the start of the campaign, every manager and vice president in the system began receiving weekly lists of their employees, notated as vaccinated, not vaccinated, or no response.” | **Value 0**; No evidence | **Value 1;** After vaccination (or attesting vaccination elsewhere), HCP were given hanging badges, stating “I’m vaccinated because I care,” to wear with their regular identification badges. Wearing the tag was not mandatory, but anyone not wearing an “I’m vaccinated” tag was required to mask while in patient care areas, regardless of their actual vaccination status. | **Value 0.33;** “Christiana Care Health System is a 2-hospital, 1,100-bed, private, not-for-profit, community-based academic healthcare system located in northern Delaware.” | **Value 0.66;** At each entrance, volunteer “clerks” (who ranged from administrative assistants to leadership personnel) scanned the HCP’s identification badge and the appropriate form (taking ~30 seconds), and then directed him/her to the next available vaccinator (volunteer nurses and pharmacists). ... Health system leadership approved use of the employee influenza vaccination rate as 1 of 3 metrics comprising a pre-existing employee bonus program, known as the Transformation Rewards Program (TRP). |
| **Frenzel** | **0.21** | **94** | **0.66** | **Value: 1;** Non-sanction based campaigns initially before mandatory: “the employee influenza vaccination program consisted of large, on-site influenza vaccination clinics that were distributed throughout >20 geographically dispersed patient care areas and research and administration buildings and were supplemented by 1 week of roaming vaccination services via mobile carts… in 2009, we piloted the mandatory participation influenza prevention program, which targeted HCWs in high-risk areas and in the nursing staff as subsequently defined” | **Value: 1**; “We expanded our education and communication campaign by prominently advertising the expanded clinic schedule and centralized, hospital-based locations and distributing various educational materials on the safety and efficacy of influenza vaccination.” | **Value: 0.66**; Managers monitored compliance “Compliance with mask use for unvaccinated HCWs was the responsibility of supervisors in each clinical area and was documented in a vaccine preventable diseases policy compliance-monitoring database” | **Value 0**; No evidence | **Value 0.66;** “compliance stickers also promoted positive reinforcement from co-workers and patients who perceived vaccination as an important patient safety measure.” | **Value 0.33**; “The University of Texas MD Anderson Cancer Center is a 656-bed National Cancer Institute–designated comprehensive cancer center with >19,000 employees.” | **Value 1;**” Senior leadership supported our initiative by aligning institutional goals with the 2007 Joint Commission requirement to increase HCW influenza vaccination rates.” |
| **Ksienski (a)** | **0.44** | **74** | **0.33** | **Value: 0;** No evidence of activity before hard mandate imposed | **Value 0**; No evidence of substantial education measures alongside punitive measures | **Value: 1**; Employees encouraged to inform on one another “HCWs who witness any colleagues violating the Policy are required to report the incident to their supervisor” | **Value 0**; No evidence | **Value 0.66**; Green dot stickers used to publically indicate vaccination status | **Value 1;** “province-wide Influenza Prevention Policy, whose primary objective is to increase vaccination coverage rates of HCWs.” | **Value 0;** not stated |
| **Ksienski (b)** | **0.57** | **75** | **0.33** | **Value: 0;** No evidence of activity before hard mandate imposed | **Value 0**; No evidence of substantial education measures alongside punitive measures | **Value: 1**; Employees encouraged to inform on one another “HCWs who witness any colleagues violating the Policy are required to report the incident to their supervisor” | **Value 0**; No evidence | **Value 0.66**; Green dot stickers used to publically indicate vaccination status | **Value 1;** “province-wide Influenza Prevention Policy, whose primary objective is to increase vaccination coverage rates of HCWs.” | **Value 0;** not stated |
| **Leibu and Maslow** | **0.22** | **94.7** | **0.66** | **Value: 0;** No evidence of activity before hard mandate imposed | **Value 0**; No evidence of substantial educational activities that could address employee concerns | **Value: 0**; No evidence | **Value 0**; No evidence | **Value 0;** Nothing mentioned | **Value 0.66;** “AHS comprised three acute care adult hospitals, a children’s hospital, an inpatient rehabilitation hospital, home care, transportation services, and several off-site clinical office practices including diagnostic facilities.” | **Value 0;** not stated |
| **Podscervinsci** | **0.42** | **96** | **0.33** | **Value: 1;** Non-sanction based campaigns initially before mandatory measures (note different intervention strategies had been implemented – evidence of one provided): “vaccine availability was advertised via multiple modalities at the center, including; mass emails, newsletter articles, and intranet postings. All employees were required to either be vaccinated or to complete a one-page signed declination form acknowledging that they understood the risks of declining the vaccine in a setting with such high-risk patients” | **Value 0**; Education only followed after declination as a penalty: “required decliners to complete enhanced influenza vaccine education” | **Value: 0.66**; Managers monitored compliance “Staff that did not meet campaign deadlines by either receiving or declining the vaccination were required to meet with their respective manager” | **Value 0**; No evidence | **Value 0.33;** In-person declination process “in front of occupation health, infection prevention staff” | **Value 0**; “The study was performed at a large comprehensive cancer care center…” | **Value 1;** “Center leadership support/involvement” described |
| **Rakita** | **0.05** | **98.9** | **1** | **Value: 0.66;** Non-sanction based campaigns initially before mandatory measures (note different intervention strategies had been implemented – evidence of one provided): “vaccine | **Value 1**; “In the spring of 2005, multiple focus groups of staff and managers were created to gather data on the barriers, educational deficits, and preferences in receiving information with regard to vaccinations. The campaign was organized around the information gathered during these focus-group sessions.” | **Value: 0;** No evidence as a policy across all employees | **Value 0**; No evidence | **Value 0;** Nothing mentioned | **Value 0.66;** “a tertiary care, multispecialty medical center that includes a 336-bed hospital, adjoining outpatient clinics, 7 regional clinics, and a research center, that provides residency teaching programs, and that employs approximately 400 physicians and a total of approximately 5,000 HCWs.” | **Value 1;** Intervention included “meetings with staff and leadership to answer questions; grand rounds speakers; trained advocates, or “champions,” of influenza vaccination; and one-on-one meetings with concerned staff. These champions included the president and CEO of the medical center.” |
| **Smith** | **0.08** | **97.7** | **1** | **Value: 0.66;** Non-sanction based campaigns initially before mandatory measures: During 2009 pandemic, prior to 2011 mandates, the hospital “engaged in unprecedented community and internal publicity, education, and other efforts to improve HCW influenza vaccination rates” | **Value 1**; “engaged in unprecedented community and internal publicity, education, and other efforts to improve HCW influenza vaccination rates” | **Value: 0.66**; Managers monitored compliance “Managers were responsible for monitoring the vaccination/exemption status of employees in their department” | **Value 0**; No structured process for encouraging interactions invited: “Aurora has a formal process for measuring HCW job satisfaction, but the process did not include questions about the vaccination policy.” | **Value 0;** Nothing mentioned | **Value 0.66;** “Aurora Health Care (Aurora) is a large integrated delivery system in eastern Wisconsin/northern Illinois that serves over 1.2 million patients per year and has over 30,000 employees....BJC Healthcare, a large Midwestern health care organization similar in size and revenue to Aurora” | **Value 1** “Senior leadership support was critical to the program’s success and its continuation.” |
| **Stuart** | **0.14** | **92.8** | **1** | **Value: 1;** Incentive-based programme implemented before a sanction-based programme was trialled before hard mandate intervention: “The program is free and incorporates mobile rounds, extended hours and promotion via newsletters and announcements. …. In December 2012, the DN was informed that to increase influenza vaccination rates, unvaccinated HCWs would be asked to wear a surgical mask during patient care throughout the influenza season. Staff were given the opportunity to ask questions about the program and raise any concerns. In February 2013, a follow-up letter confirmed that the program would be enforced, and vaccination commenced in April 2013 (when the vaccine became available). | **Value 0**; “No evidence of substantial educational activities” | **Value: 0**; No evidence | **Value 1**; “Staff were given the opportunity to ask questions about the program and raise any concerns.” | **Value 0;** Nothing mentioned | **Value 0.33**; “Monash Health is a tertiary referral service in Melbourne, Australia, with 2200 beds and 13 389 HCWs. The service provides for 1.3 million residents.” Note intervention described as being carried out in one department; unclear how many HCWs involved. | **Value 1;** Senior leaders on authorship team |

Supplementary Table 6: Soft mandate/other data table with evidence

|  |  |  |  | **‘Don’t go in cold’ (Have there been other previous vaccination campaigns prior to current intervention to change behaviours)** | **Was education provided to improve knowledge among HCW of vaccination benefits and risks?** | **Were Healthcare Workers encouraged to inform on others?** | **Two-way engagement/bidirectional communication** | **Stigma** | **Area policies implemented across institutions** | **Leading from the front – did senior staff engage in the campaign** | **Letter** |
| --- | --- | --- | --- | --- | --- | --- | --- | --- | --- | --- | --- |
| **Studies** | **Risk Ratio** | **Percentage of HCWs vaccinated at the end of intervention** | **Outcome set value** | **0 – No previous campaign or coordinated effort mentioned (vaccine may have been made available only)**  **1 – Sanction-based or accountability-based efforts or incentivisation and health promotion based efforts or combination** | **0 – No education provided or education or education did not improve levels of knowledge**  **1 – Education provided that improved knowledge of the vaccine** | **1 – Employees encouraged to inform on colleagues**  **0 – No evidence of that employees expected to inform or monitor colleagues’ vaccination status** | **0 - not stated**  **1 - formal processes for two way engagement established in the design or implementation of the intervention** | **0 – not stated**  **1 – public display of vaccination status enforced and/or stigmatising language** | **0 – single institution or modest number of institutions (<20) and/or HCWs (<10,000)**  **1 – Area based interventions (e.g. health authorities, states or counties)** | **0 – not stated**  **1 –leadership engaged in and publically voice support for vaccination campaign OR described as being instrumental for success** | **0 – Not a letter to the editor**  **1 – Letter to the editor with limited description** |
| **Camarago (Note quoted text is translated from original Spanish)** | **0.97** | **26.5%** | **0** | **Value 0;** value of 0 allocated because previous year activities described as business as usual: ”The objective of this work is to describe the results obtained in the vaccination campaign against influenza in health personnel of the season 2011-2012, in which the measures to achieve coverage, and compare them with the results of the campaign 2010-2011, which was carried out with the usual strategies.” | **Value 1;** “Information leaflet contains - Questions and answers about the flu” | **Value: 0**; No evidence | **Value 0**; Nothing stated | **Value 0**; No evidence of stigmatising processes | **Value 0;** Observational study in a tertiary hospital with a staff of approximately 3,100 workers.” | **Value 0**; Not stated | **Value 0 –** Not a letter to the editor |
| **Dey (Primary Care Teams)** | **0.99** | **21.9%** | **0** | **Value 0;** No evidence presented | **Value 1;** “The offer was made in a letter from the Consultant in Communicable Disease Control, which set out the benefits of vaccination…staff were visited by a public health nurse who raised awareness of the campaign, emphasized the safety and efficacy of the vaccination, outlined possible side effects and contraindications, discussed the impact of influenza on absenteeism, and attempted to ally anxieties and correct misconceptions.“ | **Value: 0**; No evidence | **Value 1;** Visits by nurse educator provided opportunity for two way engagement “Visited by a public health nurse who raised awareness of the campaign, emphasized the safety and efficacy of the vaccination, outlined possible side effects and contraindications, discussed the impact of influenza on absenteeism, and attempted to ally anxieties and correct misconceptions.” | **Value 0**; No evidence of stigmatising processes | **Value 1;** All worksites in a Health Authority were randomised | **Value 0;** No clear evidence of leadership practices being implemented | **Value 0 –** Not a letter to the editor |
| **Dey b (Nursing Homes)** | **0.95** | **10.2%** | **0** | **Value 0;** No evidence presented | **Value 1;** “The offer was made in a letter from the Consultant in Communicable Disease Control, which set out the benefits of vaccination…staff were visited by a public health nurse who raised awareness of the campaign, emphasized the safety and efficacy of the vaccination, outlined possible side effects and contraindications, discussed the impact of influenza on absenteeism, and attempted to ally anxieties and correct misconceptions.“ | **Value: 0**; No evidence | **Value 1;** Visits by nurse educator provided opportunity for two way engagement “Visited by a public health nurse who raised awareness of the campaign, emphasized the safety and efficacy of the vaccination, outlined possible side effects and contraindications, discussed the impact of influenza on absenteeism, and attempted to ally anxieties and correct misconceptions.” | **Value 0**; No evidence of stigmatising processes | **Value 1;** All worksites in a Health Authority were randomised | **Value 0;** No clear evidence of leadership practices being implemented | **Value 0 –** Not a letter to the editor |
| **Doratoraj (letter)** | **0.98** | **39%** | **0** | **Value 1**; Previous efforts described: “usual multi-factored approach (e.g., educational posters, newsletters, t-shirts, buttons, department meetings, and open access for long hours at multiple influenza shot stations), which had been successfully used in previous years” | **Value 1;** In addition to “no additional intervention beyond the usual multi-factored approach (e.g., educational posters, newsletters, t- shirts, buttons, department meetings, and open access for long hours at multiple influenza shot stations), which had been successfully used in previous years;” the intervention included “an influenza vaccine educational letter with the hospital logo from the head of infectious diseases” | **Value: 0**; No evidence | **Value 0**; department meetings - but not specifically about intervention and part of control condition | **Value 0**; No evidence of stigmatising processes | **Value 0;** “Eligible study participants consisted of 6723 physicians and nurses with predominantly direct patient contact at an urban tertiary care hospital.” | **Value 0;** No clear evidence of leadership practices being implemented beyond a logo included on the letter | **Value 0 –** Not a letter to the editor |
| **Doratoraj (incentives)** | **0.95** | **42%** | **0** | **Value 1**; Previous efforts described: “usual multifactored approach (eg, educational posters, newsletters, t shirts, buttons, department meetings, and open access for long hours at multiple influenza shot stations), which had been successfully used in previous years” | **Value 0;** “In addition to no additional intervention beyond the usual multi-factored approach (e.g., educational posters, newsletters, t- shirts, buttons, department meetings, and open access for long hours at multiple influenza shot stations), which had been successfully used in previous year” the intervention included “a palm tree-decorated raffle ticket offer to win a $3000 Caribbean vacation for 2, with documentation of receiving influenza vaccine.” | **Value: 0**; No evidence | **Value 0**; department meetings - but not specifically about intervention and part of control condition | **Value 0**; No evidence of stigmatising processes | **Value 0;** “Eligible study participants consisted of 6723 physicians and nurses with predominantly direct patient contact at an urban tertiary care hospital.” | **Value 0;** No clear evidence of leadership practices being implemented | **Value 0 –** Not a letter to the editor |
| **Guanche Garcel** | **0.24** | **93.2%** | **1** | **Value 1;** “compared with the previous campaign (2013–2014), the new interventions…” | **Value 1;** “Group educational sessions were conducted before the initiation of the campaign.” | **Value: 0**; No evidence | **Value 1;** Group educational sessions (implies opportunity) | **Value 0**; No evidence of stigmatising processes | **Value 0;** “At the Cuban Hospital, Dukhan, Qatar, a 75-bed secondary care center” | **Value 1;** “During our intervention, we received the full commitment of the leaders and heads of departments; that was an important advantage to achieve the results.” | **Value 1:** Letter with data |
| **Heinrich** | **0.45** | **80.3%** | **1** | **Value 1**; ”Annually, mass vaccination days are held at each campus and are supported by mobile immunisation services.” | **Value 1;** “Information regarding staff influenza vaccination sessions was provided in weekly electronic communiqués ….” | **Value 1;** “On a weekly basis, names of those staff yet to declare their intention for influenza vaccination were extracted and submitted to managers so they could prompt staff” | **Value 1;** “various hospital-wide meetings.” (implies opportunity) | **Value 1**; “A small campaign sticker was developed for placement on staff identification badges of vaccinated HCWs so that nurse immunisers could quickly identify those staff who had already received influenza vaccine.” | **Value 0**; “Alfred Health is a tertiary referral health service in Melbourne, Australia with approximately 7000 staff employed across three campuses” | **Value 1**; “feature of our infection prevention activities is the strong support of senior hospital executive and senior medical staff.” | **Value 0**: Research article published |
| **Honda** | **0.24** |  | **1** | **Value 1**; Influenza Vaccination Strategies before soft mandate intervention “Before this intervention, influenza vaccination for HCWs was voluntary.” | **Value 0**; None stated - purely information about campaign - not justification for campaign | **Value: 0**; No evidence | **Value 1;** “HCWs who submitted the declination form without documenting the primary reason were contacted by phone to obtain their reasons” (implies opportunity) | **Value 0**; No evidence of stigmatising processes | **Value 0**; Healthcare workers at a 550-bed, tertiary care, academic medical center in Sapporo, Japan | **Value 1;** Reflections from authors: “Implementing these strategies, however, required strong leadership at the institutional level, with increased recognition of the importance of vaccination of HCWs by the institution and financial support.” | **Value 0**: Research article published |
| **Lavela** | **0.49** | **77.4%** | **1** | **Value 1;** Study described a number of pre-implementation measures taken to ensure the design of the intervention was reflective of input from key stakeholders | **Value 1;** “At each site, kick-off efforts included local informational sessions for HCWs” | **Value: 0**; No evidence | **Value 1;** coordinators “met with SCI/D staff to describe the DFP and encourage participation.” (implies opportunity) | **Value 0**; No evidence of stigmatising processes | **Value 0**; Pilot intervention for “influenza vaccination of HCWs working at 2 VA spinal cord injury (SCI) centers” | **Value 1;** The intervention is described as being supported by leadership; local leadership met with staff to encourage participation | **Value 0**: Research article published |
| **Leitmeyer** | **0.96** | **26%** | **0** | **Value 0**; Baseline study conducted on reasons for low uptake but no activities that could influence implementation described e.g. building relationships or undertaking previous campaign. | **Value 1;** “The main activity of the campaign was a mass mailing to the hospitals’ medical services of all German hospitals (n∼2000), which included information and training materials, such as a PowerPoint presentation for in-house education, posters, handouts, text suggestions for employee mailings and a list of suggested activities to increase influenza vaccination among HCW.” | **Value: 0**; No evidence | **Value 0;** Comms one way only “The main activity of the campaign was a mass mailing to the hospitals’ medical services" | **Value 0**; No evidence of stigmatising processes | **Value 1**; A nationwide campaign in Germany | **Value 0;** not stated | **Value 0**: Research article published |
| **LeMaitre** | **0.44** | **69.9%** | **1** | **Value 1;** “In the intervention arm, a promotional campaign based on posters, leaflets, and an information meeting with the study team between September 15 and October 31, 2006, first sensitized staff to the benefits of influenza vaccination.” | **Value 1;** “The campaign described the potential benefits of influenza vaccination for one’s own protection and that of the residents” | **Value: 0**; No evidence | **Value 1**; Opportunities provided “Influenza vaccination was further recommended during face-to-face interviews with each member of staff present in the nursing homes between November 6 and December 15, 2006. The study team individually met all administrative staff, technicians, and caregivers to invite them to participate, and volunteers were vaccinated at the end of the interview. During the interview, prior vaccination status and, if appropriate, the reason for non-vaccination were also collected.” | **Value 0**; No evidence of stigmatising processes | **Value 1**; “Forty nursing homes matched for size, staff vaccination coverage during the previous season, and resident disability index.” | **Value 1**; Permission sought from leaders of each Nursing Home indicating leadership commitment and involvement “Each of these 376 nursing homes was sent a written invitation to participate, and 88 responded positively. Of these, 40 nursing homes in which the staff influenza vaccination coverage rate was less than 40% during the 2005/06 winter season were selected.” | **Value 0**: Research article published |
| **Lopes** | **0.59** | **45%** | **1** | **Value 1;** “Since 1999, annual influenza vaccination has been offered free of charge to all HCWs at the hospital's Immunization Center during working hours. Under this strategy, 1,202 HCWs (6% of the target population) were vaccinated in 2004, and 1,292 (6.5%) were vaccinated in 2005.” | **Value 1;** Educational campaign and a vaccination campaign: “The educational campaign addressed influenza and emphasized the importance and safety of vaccination through lectures, informal handouts, fact sheets distributed with employees' paychecks, and posters.” | **Value: 0**; No evidence | **Value 0;** One-way communication only described | **Value 0**; No evidence of stigmatising processes | **Value 0;** Single hospital involved | **Value 1;** “Both the institutional commitment to improve the rates and the involvement of employees were essential.” | **Value 1:** Letter with data |
| **Ribner** | **0.59** | **66.5%** | **1** | **Value 1; “**Before the 2006-2007 season, employees were encouraged to receive influenza vaccination, through the use of posters and articles in various employee communications. “ | **Value 1;** In addition to promotional materials made available, the declination form also included a short statement that summarized the advantages of employee vaccination. | **Value: 0**; No evidence | **Value 0;** Although employees could voice concerns it wasn’t clear how these we responded to “…the declination section of the form allowed employees to mark the reason(s) for declination of influenza vaccination. A blank space was available for employees to write in any reason(s) not preprinted on the form.” | **Value 0**; No evidence of stigmatising processes | **Value 0**; “2 adult, tertiary care, urban hospitals” | **Value 1;** “Top management took a much more public stance in support of the program, supervisors were given weekly feedback on the participation of employees in their sections, and a very popular T-shirt was given to employees who received vaccinations.“ | **Value 0**: Research article published |
| **Rothan-Tondeur (Education only)** | **0.97** | **34%** | **0** | **Value 0;** The study reports on two intervention modes; the first is reported here with no preliminary steps described | **Value 1;** After understanding reasons for declining the vaccination among HCWs, the intervention involved providing “information that would clear up all their fears and doubts and develop their altruism (HCW flu vaccination having a beneficial effect on their elderly patients).” | **Value: 0**; No evidence | **Value 0;** Reasons for declination were incorporated into the intervention but no evidence that the intervention involved two-way dialogue. Educational sessions described in didactic ways and involve lengthy presentation slides rather than opportunities for question and answer: “The slide show, entitled “Myths and Reality about Flu Vaccination”, was shown during the information sessions. The 52 slides were intended to expose myths to realities: for example, the myth that “the vaccine can cause flu” | **Value 0**; No evidence of stigmatising processes | **Value 1:** “43 health care settings” | **Value 1;** Permission sought from leaders of each Nursing Home indicating leadership commitment and involvement “A call for participation was carried out in long-term care facilities and rehabilitation care units throughout France. Department heads wishing their HCS to participate in the study designated a local investigator and contacted ORIG.” | **Value 0**: Research article published |
| **Sadlier** | **0.06** | **97%** | **1** | **Value 1;** Previous campaigns indicated “Despite successes of the outpatient vaccine programme, influenza vaccine uptake in HCWs in GUIDE in 2011-2012 was only 52% (31/60). A staff survey was undertaken in April 2012 to investigate reasons for poor vaccine uptake. Here we report results of the survey and describe interventions employed to improve vaccine uptake.” | **Value 1;** “Targeted education interventions outlining survey findings along with benefits of influenza vaccine were undertaken at departmental meetings.” | **Value: 0**; No evidence | **Value 1**; Interventions were targeted and responsive to staff concerns | **Value 0**; No evidence of stigmatising processes | **Value 0**; Single department in a hospital | **Value 0**; not stated | **Value 1:** Letter with data |
| **Shannon** | **0.59** | **44%** | **1** | **Value 1**; Evidence of previous campaign activity: “have traditionally been low-around 5% in recent years. When vaccinations have been offered, hospital staff have frequently said, "The flu shot makes me sick," "I never get the flu," or "I don't trust it."” | **Value 1; “**While conducting annual infection control in-service education presentations in various departments, the infection control coordinator offered vaccinations” | **Value: 0**; No evidence | **Value 0;** Not stated | **Value 0**; No evidence of stigmatising processes | **Value 0**; Single hospital | **Value 0;** Nothing stated | **Value 1:** Letter with data |
| **Smedley** | **0.98** | **5%** | **0** | **Value 1;** vaccine offered routinely to employees since early 1990s - not promoted actively but promoted in advance of intervention | **Value 1 ;** Intervention involved distribution of a leaflet describing effectiveness of vaccine and a short presentation on influenza vaccine | **Value: 0**; No evidence | **Value 1**; Intervention was targeted and responsive to staff concerns | **Value 0**; No evidence of stigmatising processes | **Value 0**; Single hospital trust unit | **Value 0**; discussion includes reference that gaining support of senior medical managers and clinical role models might improve vaccine uptake, although not described in present intervention | **Value 0**: Research article published |
| **Thomas** | **0.59** | **54%** | **1** | **Value 1**; An educational intervention began 1 month before vaccination available | **Value 1**; An educational intervention (no further description) | **Value: 0**; No evidence | **Value 1;** Individual encouragement and answering of questions was offered | **Value 0**; No evidence of stigmatising processes | **Value 0**; Single care setting | **Value 1**; A key element of programme involved immunising physicians in presence of other staff - including medical director | **Value 0**: Research article published |
| **Zimmerman incentives** | **0.97** | **38.4% (based on staff with direct patient contact)** | **0** | **Value 1;** Previous campaigns evaluated and used to plan current intervention | **Value 1**; Materials produced “that addressed myths about influenza, the vaccine, and motivations for choosing to be vaccinated or not” | **Value: 0**; No evidence | **Value: 0**; No evidence | **Value 0**; No evidence of stigmatising processes | **Value: 0**; Eleven facilities included | **Value: 0**; No evidence | **Value 0**: Research article published |
| **Zimmerman increased access (carts)** | **0.97** | **39.0% (based on staff with direct patient contact)** | **0** | **Value 1;** Previous campaigns evaluated and used to plan current intervention | **Value 1**; Materials produced “that addressed myths about influenza, the vaccine, and motivations for choosing to be vaccinated or not” | **Value: 0**; No evidence | **Value: 0**; No evidence | **Value 0**; No evidence of stigmatising processes | **Value: 0**; Eleven facilities included | **Value: 0**; No evidence | **Value 0**: Research article published |
